# Fully exploratory network independent component analysis of the 1000 functional connectomes database

**DOI:** 10.3389/fnhum.2012.00301

**Published:** 2012-11-06

**Authors:** Klaudius Kalcher, Wolfgang Huf, Roland N. Boubela, Peter Filzmoser, Lukas Pezawas, Bharat Biswal, Siegfried Kasper, Ewald Moser, Christian Windischberger

**Affiliations:** ^1^MR Centre of Excellence, Center for Medical Physics and Biomedical Engineering, Medical University of ViennaVienna, Austria; ^2^Department of Statistics and Probability Theory, Vienna University of TechnologyVienna, Austria; ^3^Department of Psychiatry and Psychotherapy, Medical University of ViennaVienna, Austria; ^4^Department of Radiology, University of Medicine and Dentistry of New Jersey, New Jersey Medical SchoolNewark, NJ, USA

**Keywords:** magnetic resonance imaging, fMRI, resting-state, ICA, default-mode network

## Abstract

The 1000 Functional Connectomes Project is a collection of resting-state fMRI datasets from more than 1000 subjects acquired in more than 30 independent studies from around the globe. This large, heterogeneous sample of resting-state data offers the unique opportunity to study the consistencies of resting-state networks at both subject and study level. In extension to the seminal paper by Biswal et al. (2010), where a repeated temporal concatenation group independent component analysis (ICA) approach on reduced subsets (using 20 as a pre-specified number of components) was used due to computational resource limitations, we herein apply Fully Exploratory Network ICA (FENICA) to 1000 single-subject independent component analyses. This, along with the possibility of using datasets of different lengths without truncation, enabled us to benefit from the full dataset available, thereby obtaining 16 networks consistent over the whole group of 1000 subjects. Furthermore, we demonstrated that the most consistent among these networks at both subject and study level matched networks most often reported in the literature, and found additional components emerging in prefrontal and parietal areas. Finally, we identified the influence of scan duration on the number of components as a source of heterogeneity between studies.

## Introduction

Since the seminal report by Biswal et al. (1995), low-frequency spontaneous fluctuations (in the range of 0.01–0.1 Hz) of blood oxygen level dependent (BOLD) signal in the brain have consistently been found in the absence of task-induced activity. Research in this area has increasingly gained momentum during the last years, from both a methodological perspective (Margulies et al., 2010) and a neuroscientific point of view (Raichle and Snyder, 2007). Even beyond the original finding of the motor network by Biswal et al. (1995), an increasing number of other networks have consistently been reported, and these are now referred to as resting state networks (Fox and Raichle, 2007). Most commonly, networks related to motor function, visual processing, executive function, auditory processing, memory, as well as the default-mode network have been named in this context (Damoiseaux et al., 2006; Robinson et al., 2009; Schöpf et al., 2010). However, variability in the exact extent of networks reported as well as the total number of resting-state networks and their possible subdivisions still exist today (Leech et al., 2011), and a quantification of variability of number and type of resting-state networks identified in the data of different centers has not yet been performed.

In the most general terms, the concept of brain networks is based on the measure of functional connectivity, defined as temporal coherence between the low-frequency (< 0.1 Hz) BOLD signal of spatially remote brain regions (Richiardi et al., 2011). This functional connectivity is commonly calculated using a seed-based analysis approach, where temporal correlation is calculated with respect to a seed voxel or region. Consequently, a clear-cut distinction of the networks is limited by the fact that a single brain region can be involved in several networks (Joel et al., 2011), e.g., the lateral parts of the parietal lobes that are associated with both the default-mode network and the frontoparietal (working memory) network (Corbetta and Shulman, 2002). Additionally, the detection of previously unknown networks and the identification of unexpected properties are hampered by the inherent necessity for *a priori* selection of seed regions rendering seed-based functional connectivity an inherently parametric approach.

Non-parametric methods have been used to overcome this limitation, among them clustering and pattern-recognition algorithms, but it is independent component analysis (ICA)—or, more specifically, spatial ICA, as opposed to temporal ICA—that has emerged as the most successful method to identify spatially independent brain networks, as witnessed by a large number of influential studies (Damoiseaux et al., 2006; Smith et al., 2009; Biswal et al., 2010; Allen et al., 2011). The capability of ICA to identify neuroscientifically meaningful effects is corroborated by the similarity of results from ICA of resting state fMRI data and from ICA of electrophysiology data acquired using magnetoencephalography (MEG) (Brookes et al., 2011). In contrast to seed-based analysis, though, ICA offers no canonical method for group comparison, and multiple solutions have been put forward to address this question, including, among others, time-concatenated ICA (Calhoun et al., 2001), i.e., time concatenation of individual-subject time series before ICA analysis, tensor-based ICA, tensor-based probabilistic ICA (Damoiseaux et al., 2006), probabilistic ICA (PICA) (Luca et al., 2006), and self-organizing group ICA (sogICA) (van de Ven et al., 2008). Earlier studies relying on these methods have found varying numbers of independent components based on automated dimension estimation, ranging from 5 (Luca et al., 2006) to about 12 (Damoiseaux et al., 2006; Robinson et al., 2009), while more recently, the use of a pre-specified number of components has become more widespread, using either a low model order with about 20 components (Smith et al., 2009; Biswal et al., 2010) or a high model order with about 75 components (Allen et al., 2011), different choices of model order of course leading to the identification of different networks or subdivisions of networks.

The variability of results depending on model order has been investigated by Abou-Elseoud et al. (2010), who found that low model order ICA results had highest repeatability, while higher model orders lead to the identification of finer subdivisions of the networks, up to a model order of about 100—beyond that value, repeatability only declined without any additional benefits. Model order is not the only source of variability in the results of ICA studies, though. A certain amount of inconsistency between studies is due to random variability between samples, which, due to practical limitations, often comprise only 20–30 subjects. Finally, some divergence between results can be attributed to methodological issues: for once, there is the inherent stochasticity of the fastICA algorithm, the basis for most ICA implementations currently employed (Himberg et al., 2004) and additional variability may be introduced by the heterogeneity of preprocessing strategies (Weissenbacher et al., 2009).

Evaluation of between-subject variability of group components can be undertaken from two directions. Back reconstruction algorithms (Biswal et al., 2010; Allen et al., 2011, 2012) start with group components and evaluate how consistent the connectivity of these group components is on the single-subject level. In this study, we opted for the opposite direction—starting with individual-subject components and evaluating the variability of components between subjects—and chose fully exploratory network ICA (FENICA), proposed by Schöpf et al. (2010), as a means for combining single-subject results at group level. FENICA is a group ICA method that, based on single-subject ICA, calculates each group component as the mean of the most similar components, one of each individual-subject ICA. This in turn allows for the group components to be directly related to single-subject ICA components and thus to gain a more immediate view on the differences of ICA components across subjects. In addition, the averaging of components from multiple ICA runs in FENICA helps to increase stability of group results and limits the effects of the stochasticity of fastICA (Himberg et al., 2004), though some caution in this respect is still advisable when interpreting individual single-subject components.

It must be noted, though, that the heterogeneity of populations investigated by different studies leads to inter-study variability near-impossible to overcome within the scope of a single study. A comprehensive exploratory analysis should therefore neither take into account only a single population nor a single setup of scanner hardware but rather combine a large number of different datasets from different studies. A meta-analytic approach using individual-subject data therefore seems most promising to summarize available evidence about resting-state networks and to assess heterogeneity between datasets of different origin (Huf et al., 2011). The 1000 Functional Connectomes Project (Biswal et al., 2010), a collection of resting-state fMRI datasets from over 30 international centers encompassing more than 1000 different subjects, provided us with the opportunity to perform precisely this kind of analysis on a suitably broad basis for approaching the question of consistent networks on a large scale.

## Methods

The entirety of the dataset of the 1000 Functional Connectomes Project (Biswal et al., 2010) directly available at its webpage was downloaded (see http://www.nitrc.org/projects/fcon_1000). To avoid the most important sources of heterogeneity as well as complications due to non-independence, subjects with more than one run in the dataset were excluded from the analysis. The final sample consisted of 1000 subjects (age 28 ± 13, 561 females; see Table 1) randomly sampled from the remainder of the dataset consisting of 33 independent samples originating from 26 centers in North America (15), Europe (8), Asia (2), and Australia (1). The original scans were performed using echo planar imaging (EPI) during resting-state with variable scanning parameters and brain coverage at 1.5 T, 3 T, and 4 T, with a duration between 216 and 590 s.

**Table 1:** **Demographic statistics of the sample analyzed**.

	**Study**	**N**	**%Male**	**Mean age**	**SD age**	**Voxel size**	**TR**	**Volumes**	**Duration**	**Components**
1	AnnArbor_a	25	88.0	21.0	7.4	35.4	1.00	295	295.0	17
2	AnnArbor_b	36	47.2	NA	NA	37.8	1.00	395	395.0	19
3	Atlanta	28	46.4	30.9	9.9	47.3	2.02	205	414.1	16
4	Baltimore	23	34.8	29.3	5.5	21.3	2.50	123	307.5	15
5	Bangor	20	100.0	23.4	5.3	27.0	2.00	265	530.0	20
6	Beijing	198	38.4	21.2	1.8	35.2	2.00	225	450.0	17
7	Berlin	26	50.0	29.8	5.2	36.0	2.30	195	448.5	19
8	Cambridge	198	37.9	21.0	2.3	27.0	3.00	119	357.0	17
9	Cleveland	31	35.5	43.5	11.1	16.0	2.80	127	355.6	17
10	Dallas	24	50.0	42.6	20.1	47.3	2.00	115	230.0	13
11	ICBM	86	47.7	44.2	17.9	27.0	2.00	192	384.0	12
12	Leiden_2180	12	100.0	23.0	2.5	40.7	2.18	215	468.7	19
13	Leiden_2200	19	57.9	21.7	2.6	40.7	2.20	215	473.0	19
14	Leipzig	37	43.2	26.2	5.0	36.0	2.30	195	448.5	20
15	Milwaukee_a	18	NA	NA	NA	84.4	2.00	175	350.0	22
16	Milwaukee_b	46	32.6	53.6	5.8	56.2	2.00	175	350.0	16
17	Munchen	16	62.5	68.4	4.0	43.0	3.00	72	216.0	11
18	Newark	19	47.4	24.1	3.9	59.1	2.00	135	270.0	14
19	NewHaven_a	19	52.6	31.0	10.3	70.9	1.00	249	249.0	13
20	NewHaven_b	16	50.0	26.9	6.3	65.0	1.50	181	271.5	18
21	NewYork_a	25	80.0	35.0	9.6	27.0	2.00	192	384.0	13
22	NewYork_a	84	51.2	24.4	10.1	27.0	2.00	192	384.0	13
23	NewYork_b	20	40.0	29.8	9.9	36.0	2.00	175	350.0	13
24	Ontario	9	NA	NA	NA	64.0	3.00	105	315.0	15
25	Orangeburg	20	75.0	40.6	11.0	61.2	2.00	165	330.0	12
26	Oulu	103	35.9	21.5	0.6	70.4	1.80	245	441.0	15
27	Oxford	22	54.5	29.0	3.8	31.5	2.00	175	350.0	17
28	PaloAlto	17	11.8	32.5	8.1	57.9	2.00	235	470.0	20
29	Pittsburgh	17	58.8	37.9	9.0	31.3	1.50	275	412.5	13
30	Queensland	19	57.9	25.9	3.9	46.5	2.10	190	399.0	17
31	SaintLouis	31	45.2	25.1	2.3	64.0	2.50	127	317.5	17
32	Taipei_a	13	NA	NA	NA	56.3	2.00	295	590.0	25
33	Taipei_b	8	NA	NA	NA	47.3	2.00	175	350.0	17

Mean and standard deviation of age are given in years, voxel size in mm^3^, TR in seconds; the column Volumes lists the number of volumes (or time points) scanned for every subject in the study, the column Duration lists scan duration in seconds and the column Components contains the median number of ICA components identified for the subjects of this study.

Due to the *post-hoc* nature of the 1000 Functional Connectomes dataset's formation by merging independent, non-coordinated individual studies, between-study heterogeneity is an important issue to clarify before analyzing this dataset. The original analysis by Biswal et al. (2010) has, as one of its main results, established the feasibility of using the dataset as a whole with a reasonable expectation to obtain homogeneous results, even for studies using scanners with different magnetic field strength. Further attempts to include estimated study quality, e.g., for weighting purposes, are discouraged in the meta-analytic setting due to possible bias introduced by such procedures (Huf et al., 2011), and are thus not part of our analysis.

Preprocessing of the resting-state fMRI data was performed according to Weissenbacher et al. (2009) by first applying motion correction and spatial smoothing using an 8 mm FWHM Gaussian kernel followed by correction for mean cerebro-spinal fluid (CSF), white matter (WM) and gray matter signals as well as motion parameters. Subsequently, time series were filtered using a bandpass of the interval 0.01–0.1 Hz, and ICA was calculated on the resulting time series using FSL MELODIC (Smith et al., 2004) with the dimension estimation criterion LAP, yielding a number of components in the range of typical low model order studies. Automated model order estimation rather than fixed model order was chosen to allow for a comparison of model order estimates between subjects and between the datasets of the individual studies, as well as for an estimation of the variability of these estimates. Finally, preprocessing was concluded by normalization to MNI 152 standard space and re-sampling to 3 mm isotropic voxels to enable group level analyses. All preprocessing steps were computed using AFNI (Cox, 1996), second-level analyses were performed in R 2.13.1 (R Development Core Team, 2012), using specialized packages for fMRI analysis, parallelization, and handling of large data (Tabelow et al., 2011; Boubela et al., 2012).

Following this preprocessing, individual-subject ICA results—one z-map for each component—were combined using the FENICA algorithm proposed by Schöpf et al. (2010). Briefly, the algorithm aims at exploratorily finding components consistent over a population of subjects and is composed of three stages: (1) identification of pairs of matching maps, (2) building of candidate average maps, and (3) selection of final average maps.

To allow for automated and thus reproducible exploratory selection of parameters of the algorithm, two modifications to the algorithm as originally described (Schöpf et al., 2010) have been made to adapt it to the necessities of the large dataset while minimizing the influence of observer bias (Boubela et al., 2012). First, identification of eligible pairs was set to match the number of original components. Second, the similarity threshold (Schöpf et al., 2010) to discard average components similar to at least one other component with a higher *t*-sum was chosen as the lowest value that produced a number of final components corresponding to the median number of components of the individual-subject results. Related groups of final components were defined by spatially clustering the components using hierarchical clustering with centroid distance between clusters (Mangiameli et al., 1996) using Kolmogorov–Smirnov distance (Kolmogorov, 1933) between *z*-values of components as the distance between maps.

Consistency across subjects was assessed for each resulting component by calculating the correlation of the original pairwise average map that was used as the candidate for the generation of the group map with each subject's respective best matching component. The distribution of these correlation coefficients was then used to evaluate the consistency of each component.

An assessment of spectral characteristics of group networks was performed by computing individual-subject power spectra for each group network using a back reconstruction algorithm. The individual-subject spectra were averaged to determine power spectra at group level. From these, the dynamic range (i.e., the difference between the power at the peak of the spectrum and the minimum power of frequencies higher than this peak) and the power ratio (i.e., the ratio between the integral of the power of the frequencies below 0.1 Hz and the integral of the power of the frequencies higher than 0.15 Hz) are computed for each of the group components identified (Robinson et al., 2009).

In addition, the whole computation was performed separately for the subset of subjects of each individual study to determine consistency of components across studies. Group components were considered to be present in an individual study sample if and only if there was a component in the individual study results that could be partner-matched (Wang and Peterson, 2008) to that group component, i.e., if the group component had highest spatial correlation among all group components to the individual-study component in question and vice versa.

To assess the relationship between scan duration and number of components found at individual-subject level, a least-squares regression as well as a robust MM-estimator (Koller and Stahel, 2011) were fitted.

## Results

**Figure 1:**
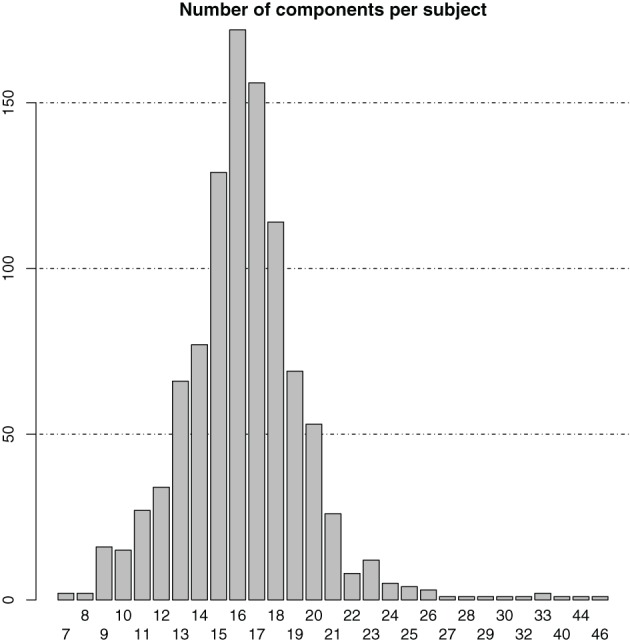
**Bar plot of the distribution of the number of components in the individual-subject ICA results**.

At single-subject level, the number of ICA components was symmetrically distributed with a mean and median number of components both equal to 16 ± 3.5 (SD) (cf. the bar plot in Figure 1). In total, there were 16,365 individual-subject components from which the same number of candidate pairs of components were selected for calculation of average maps.

**Figure 2:**
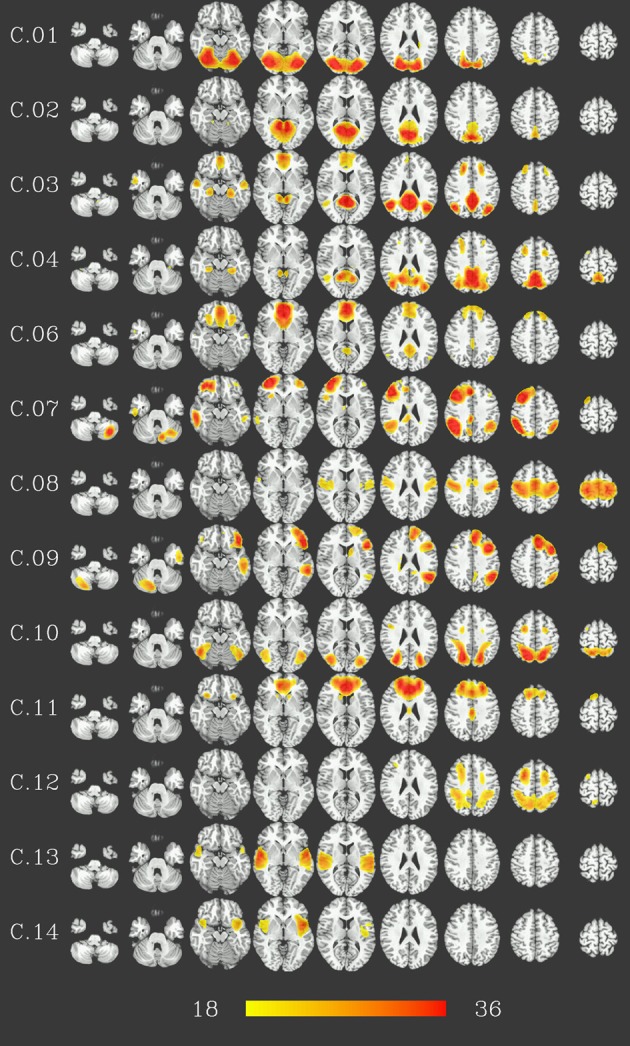
**Consistent gray-matter networks from 1000 resting-state datasets**. Results are presented as *t*-values, thresholded at *t*_999_ = 18 (*p* = 1.6 · 10^−57^, FWE-corrected), each row corresponding to one network showing nine representative slices spaced 15 mm in z direction. Red color represents highest *t*-values, images are shown in radiological convention (the right side of the brain is displayed on the left). Color bar shown for *t*-values between 18 and 36 (bottom).

**Figure 3:**
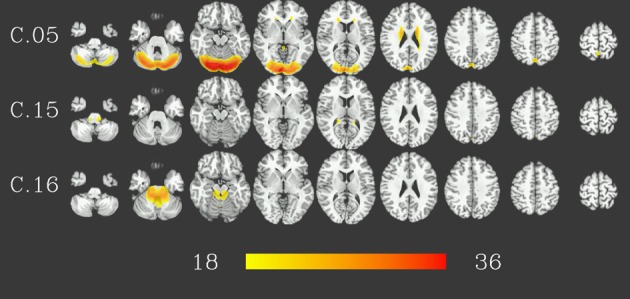
**Artifact components consistent in 1000 resting-state datasets**. Results are presented as *t*-values, thresholded at *t*_999_ = 18 (*p* = 1.6 · 10^−57^, FWE-corrected), each row corresponding to one component showing nine representative slices spaced 15 mm in z direction. Red color represents highest *t*-values, images are shown in radiological convention (the right side of the brain is displayed on the left). Color bar shown for *t*-values between 18 and 36 (bottom).

At group level, 16 group components were identified for a similarity threshold of 0.75, chosen to produce a number of components corresponding to the median number of individual-subject components as detailed above. Of these components, 13 can be described as gray matter networks (shown in Figure 2, using an arbitrary thresholded at *t*_999_ = 18, *p* = 1.6 · 10^−57^ FWE corrected for displaying purposes), and 3 show consistent activity mainly located in voxels outside the gray matter (components C.05, C.15 and C.16, see Figure 3) and will therefore be referred to as (consistent) artifact components from here on.

Gray matter networks, designated C.01 to C.16 in descending order of their voxelwise sum of *t*-values, can be described as follows (for correspondence to known resting-state networks cf. Discussion). Component C.01 corresponds mainly to the occipital lobe. Component C.02 includes the posterior cingulate cortex and precuneus. Component C.03 shows activation in ventral medial prefrontal, posterior cingulate, and lateral parietal cortex as well as hippocampus and, to a lesser extent, the inferior temporal lobe. Component C.06 is situated in ventral and dorsal medial prefrontal cortex, posterior cingulate cortex and, to a lesser extent, lateral parietal cortex. Thus, these two components correspond to regions commonly identified as part of the default-mode network, with component C.03 more focused in the posterior, and component C.06 in the anterior parts. C.04 is centered on the posterior cingulate cortex and precuneus, with co-activations in the dorsolateral prefrontal cortex. Components C.07 and C.09 are strongly lateralized, situated in the ventral and dorsal lateral prefrontal cortex, lateral parietal cortex and superior temporal lobe—predominantly right for component C.07, and left for component C.09—as well as the respective contralateral part of the cerebellum. Component C.10 and C.12 encompass dorsal parietal, precentral, as well as occipitotemporal (BA 37) areas, with C.10 being more focused on the ventral parts and C.12 more strongly involved in the dorsal parts of these areas, in particular the precentral areas. Component C.08 covers the pre- and postcentral gyri and can be described as a sensory-motor network, C.11 is focused on the anterior cingulate cortex, with co-activations in the dorsolateral prefrotal, orbitofrontal as well as posterior cingulate cortex. Finally, components C.13 and C.14 are located on the temporal lobes.

**Figure 4:**
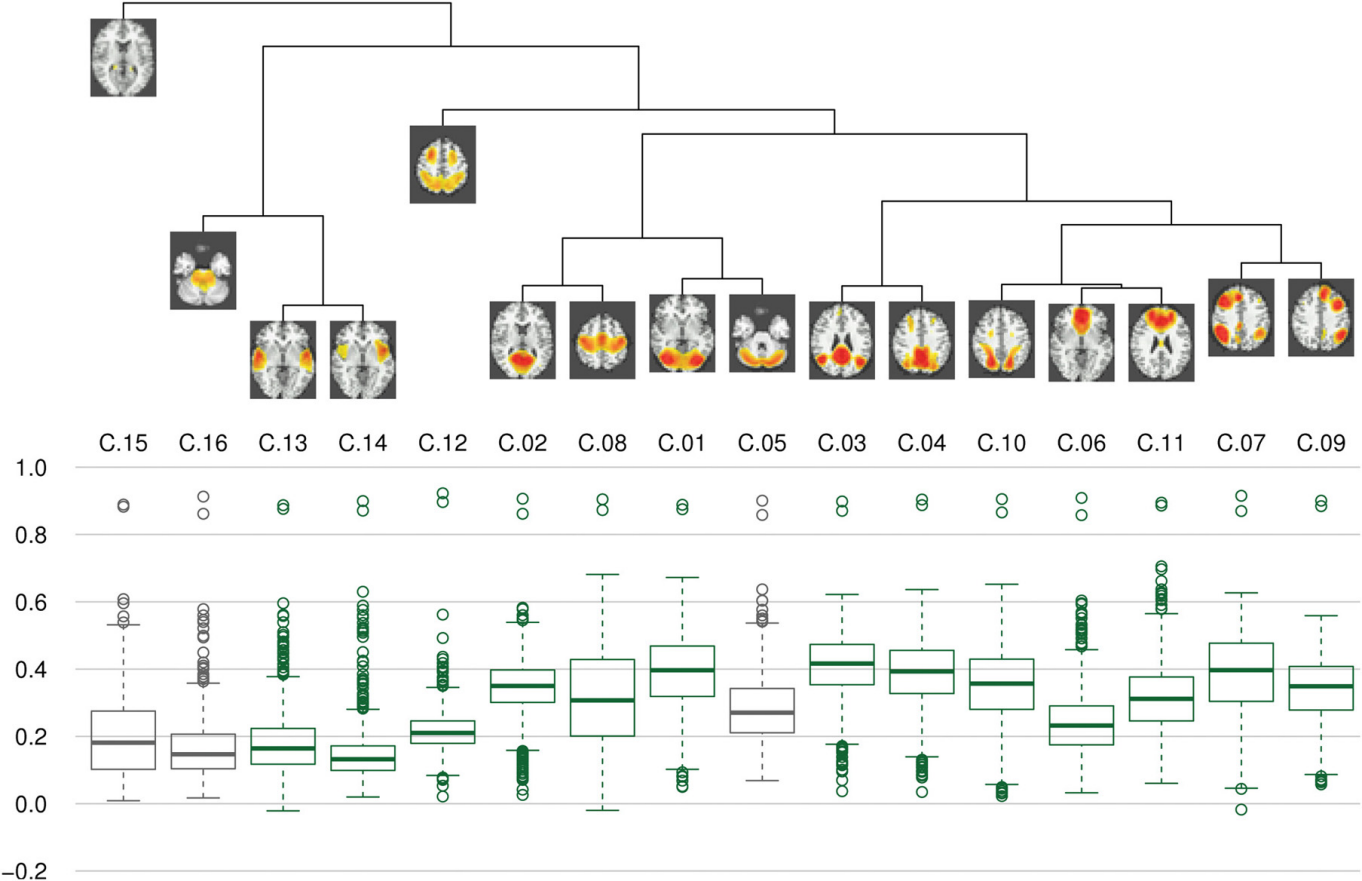
**Clustering and consistency of results**. On top, a dendrogram represents results of hierarchical clustering using complete linkage distance between clusters. Below, boxplots of correlation coefficients illustrate the consistency of components between subjects. Boxes for gray matter components are drawn in green, artifacts are drawn in dark gray. It can be seen that the clusters with lower consistency (i.e., C.12–C.16) are quite distinct from a more homogeneous cluster of higher consistency components.

Clustering results of the networks are presented as a dendrogram in Figure 4, along with boxplots of the distribution of the correlation coefficients between candidate pairwise average maps and best matching components of each subject, showing inter-subject consistency of the final component maps. It can be noted that the gray matter components (shown in green), whose correlation coefficients are mostly around 0.3–0.4, are generally more consistent than the components identified as artifacts (shown in gray), with correlation coefficients of around 0.2. Still, there is also a number of gray matter components (C.06, C.12–C.14) which show lower consistency, comparable to that of the artifact components. In addition, on the left side of the dendrogram in Figure 4, one can find the least consistent components (C.12–C.16) situated quite apart from the components with higher consistency (including C.05, the most consistent of the three artifact components).

**Figure 5:**
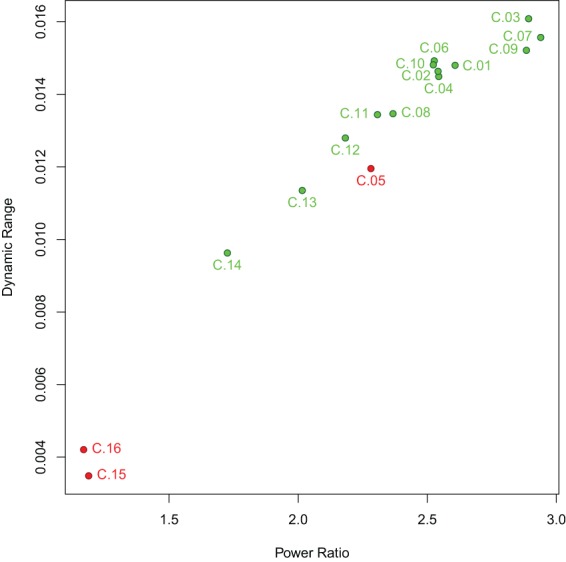
**Power ratio and dynamic range of the components identified**. Gray matter components are shown in green, artifact components in red. Both power ratio and dynamic range are highest for gray matter networks and lower for artifactual components, though with some overlap since the most consistent of the artifact components, C.05, has higher power ratio and dynamic range than the two least consistent gray matter components, C.13 and C.14.

**Figure 6:**
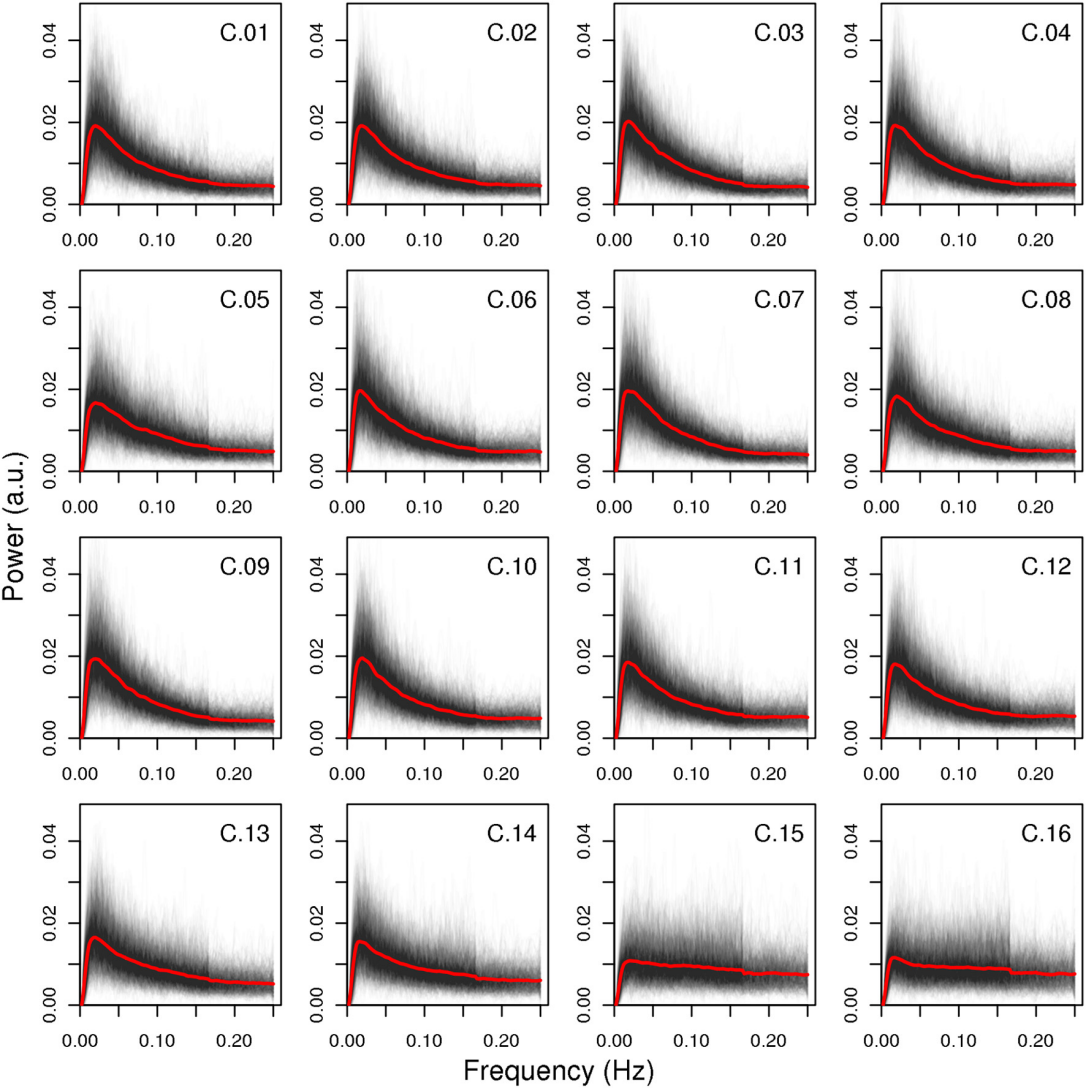
**Spectra of all components**. All single-subject spectra were drawn as black lines, and the mean spectrum of each component is displayed as a red line. The artifact components C.15 and C.16 have markedly different spectra than the other components, the third artifact C.05 is more similar to the gray matter components in that it has higher power in lower frequency bands, but its decline in power after the peak is less steep.

The spectral characteristics of the networks indicate that the artifact components have lower power ratio between high and low frequencies as well as lower dynamic range (see Figure 5): both values are lowest for components C.15 and C.16, while component C.05 shows higher values than the two least consistent gray matter components C.13 and C.14, but still lower than the other components. Indeed, the difference in the spectra for the components C.15 and C.16 is evident at the first glance (see Figure 6), while the spectrum of C.05 seems more similar to the spectra of the gray matter components. It is noteworthy here that the spectral characteristics of this occipital component can be related to an observation by Birn et al. (2008), where a medial occipital component was found to at least partly reflect respiratory-induced changes. One possible interpretation put forward by Birn et al. was that the component might be a mixture of gray matter and respiratory signal, which is consistent with our observation of the spectrum being more similar to gray matter component spectra than the other two artifact components.

**Figure 7:**
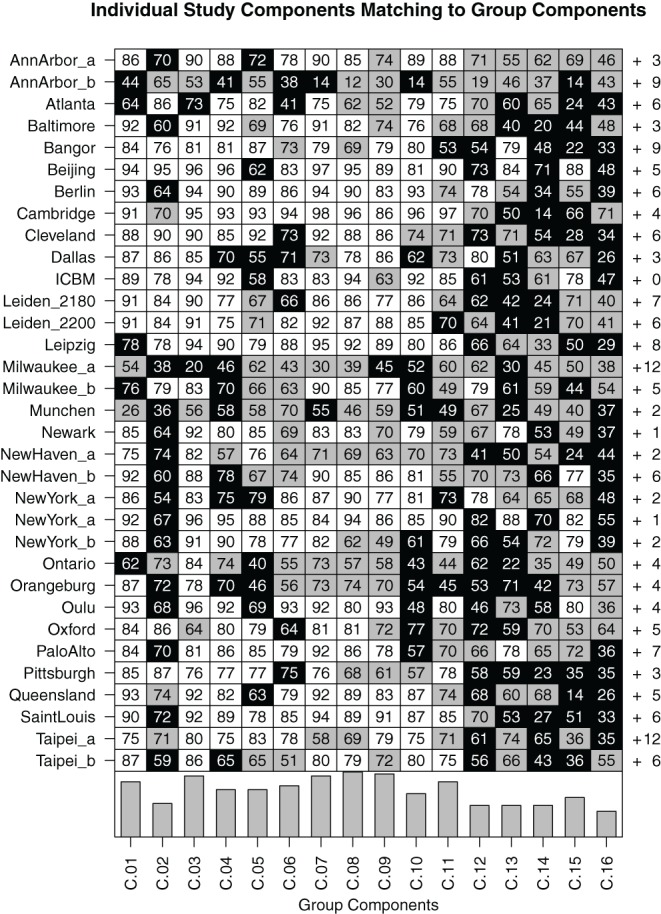
**Matrix of bidirectional 1-to-1 matching of components in individual-study analyses compared to group results from the whole dataset comprising 1000 subjects**. Each field contains the spatial correlation coefficient (multiplied by 100 for readability) between the group component and the best matching individual-study component. White fields indicate a bidirectional match with a spatial correlation of at least 0.75, gray fields indicate bidirectional matches with lower spatial correlation, and black fields indicate the absence of a bidirectional match (note that the spatial correlation to the best matching component can nonetheless be high in some cases). To the right of each row, the number of other components found in the individual-study analysis which do not bidirectionally match any of the group components is listed. At the bottom of each column, a bar plot indicates the study-level consistency of each component, counting the number of studies in which a bidirectional match was found.

At study level, Figure 7 shows comparisons between the components in the individual FENICA component sets of all sites analyzed separately with the group components from the analysis of the whole sample presented in Figures 2 and 3. The most consistent components (i.e., C.01, C.03, C.07–C.09) are characterized by the existence of a successful match in almost all individual sites as well as high spatial correlation of the best matching components with the group component.

Of note, it can be seen that while on the one hand there are some components that can be found in almost all component sets of single studies analyzed separately (C.01, C.03, C.07–C.09), other components appear only in the single-study results of about half of the studies included in the 1000 Functional Connectomes dataset (C.02, C.12–C.16). Still, even the most consistent group components do not exhibit uniformly high spatial correlation with their matching components or fail to bidirectionally match with a component from each set of single-study components. Component C.08, for instance, has a partner-matched component in every single study, yet spatial correlations with its matched components are as low as 0.12 for the dataset Ann Arbor b, 0.39 for Milwaukee a and 0.46 for München. Conversely, there is a generally low consistency of some studies with all group results (the maximum correlations of a component of the three example studies mentioned above with a group component are 0.65, 0.7, and 0.62, respectively). On the other extreme, there are some group components which could not be unambiguously matched to only one component in a given study despite there being a component with high spatial correlation. This is an indication that there might be a second equally well matching component in this study's dataset, probably due to a division of the network into subcomponents.

**Figure 8:**
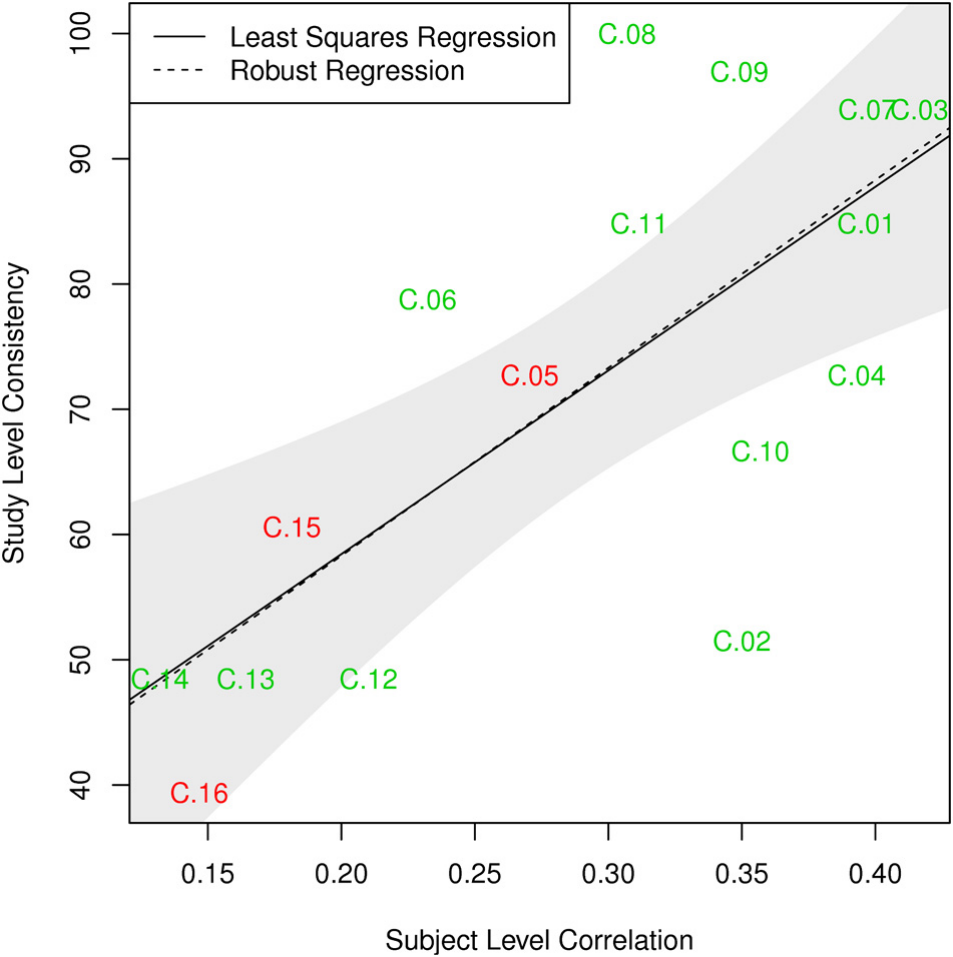
**Comparison of subject level consistency based on spatial correlation of single subject component maps with study level consistency based on identification of maps in individual-study results**. Ordinary least squares (OLS; *p* = 0.002) and robust MM-estimator (*p* < 10^−4^) illustrate the relationship between the two measures. The shaded area corresponds to a 95% confidence interval for the OLS estimator.

The components can thus be divided in three categories. First, there are components with high consistency at both single-subject and study level; these include C.01, C.03, C.04, and C.07–C.11. The second group of components can be characterized as those least consistent at both levels, notably C.12–C.16. As a third group, some components show differences in these two metrics: C.02 is about as consistent as other gray matter components at single-subject level, but can be found in only half of the single-study samples, C.05 and C.06 are among the less consistent components at single-subject level, but show average consistency at study-level. Figure 8 illustrates this relationship between subject level and study level consistency.

**Figure 9:**
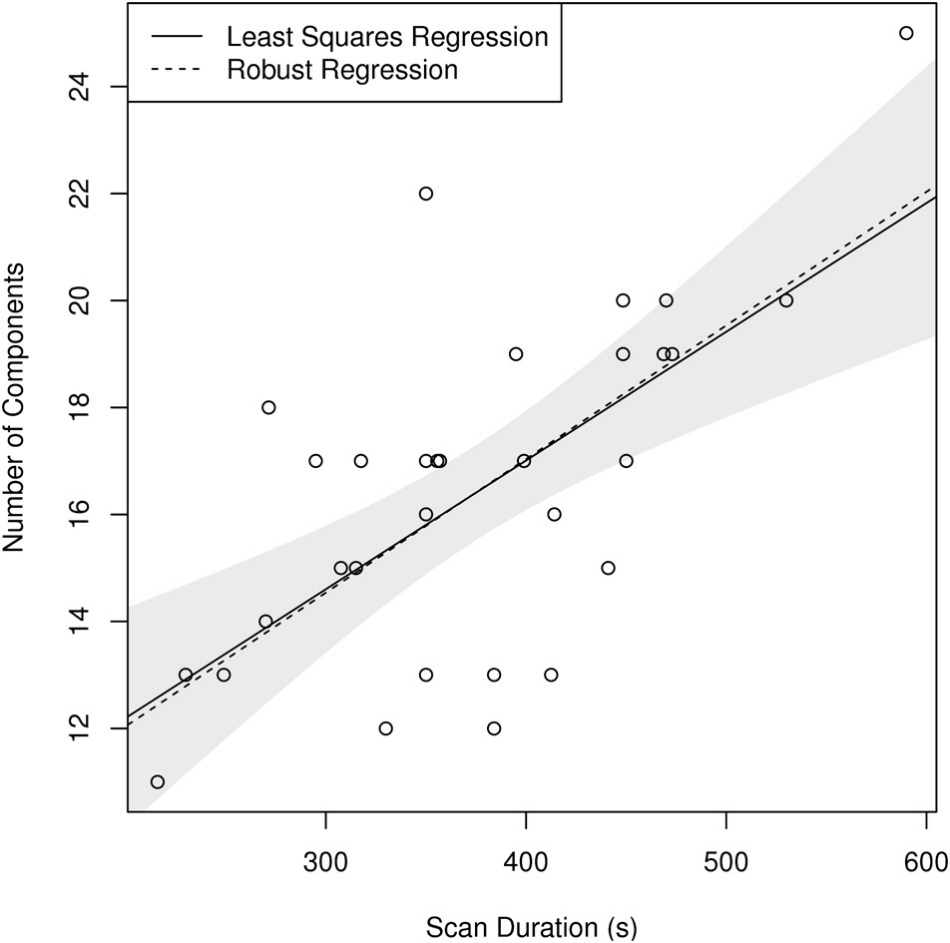
**Scatterplot showing a linear relationship between scan duration in seconds and median number of components per subject identified in each individual study using LAP criterion in FSL MELODIC**. Note that both ordinary least squares (OLS) and the robust MM-estimator identify the same relationship, highlighting the robustness of this ratio (*p* < 10^−4^ for both estimators). The shaded area corresponds to a 95% confidence interval for the OLS estimator.

Finally, single-subject results show systematic variation of the number of components, identified by MELODIC using the LAP criterion, depending on the study of origin of the individual-subject dataset (see Figure 9). In particular, there is a significant correlation between the median number of components found in the subjects of a study with the duration of the scans of that study, with longer scans being associated with larger number of components. The robustness of this finding is corroborated by the observation that the application of a robust methods of moments regression leads to the same result.

## Discussion

In this study we analyzed a publicly available dataset of 1000 subjects' resting-state scans using the exploratory analysis method FENICA (Schöpf et al., 2010; Boubela et al., 2012). Our goal was to examine the consistency of resting-state networks identified in previous, smaller studies in a very large sample originating from multiple, international centers, and to assess heterogeneity of results between studies.

Altogether, we identified 16 consistent components. Among them, 13 can be regarded as neuroscientifically meaningful gray matter components, while the remaining three may be attributed to consistent artifacts. The latter mostly correspond to ventricular/CSF regions, the most consistent of the three components being situated mainly in the occipital CSF. The consistency values of these artifacts, in particular the inter-subject spatial correlation coefficient of these component maps of around 0.2, can be seen as a reference to which the consistency of gray matter components can then be related.

Indeed, the consistency values of most gray matter networks are markedly higher than those of all artifact components. Many of the gray matter networks identified in this study correspond to networks as previously published (Damoiseaux et al., 2006; Smith et al., 2009; Biswal et al., 2010; Allen et al., 2011). The occipital visual network (C.01), the sensory-motor network (C.08) as well as the dorsal parietal network (C.10) have been reported in most fMRI studies on the resting brain. This study adds a quantification of the consistency of these networks, showing that the visual (C.01) and the sensory-motor networks (C.08) can be found in almost all single-study samples (85% for C.01 and 100% for C.08), and the dorsal parietal network (C.10) appears in two-thirds of these samples. The components C.07 and C.09, encompassing the regions associated with memory function in dorsolateral prefrontal and lateral parietal cortex, are both also among the most consistent networks identified (94% and 97%, respectively), highlighting the lateralized subdivision of the working memory network.

We found two networks associated with regions of the default-mode network of the brain (C.03 and C.06), with the posterior of the two (C.03) being one of the most consistent networks identified, appearing in 94% of single study results, and the anterior (C.06) being found in 79% of the study samples. The anterior default mode component, however, exhibits lower spatial correlation both between the group component map and study-level components as well as between the group component map and single-subject component maps. This supports the hypothesis of a functional segregation of the default-mode network (Kim and Lee, 2011), with one part particularly involved in the prefrontal regions, and the other part dominating in the posterior cingulate cortex, the parietal cortex, and the hippocampus. This division into anterior and posterior parts of the default-mode network, although not a novel concept, is not yet fully embraced in the literature (Buckner et al., 2008).

In contrast to the high consistency in the subdivision of the working memory networks in a left and right part, there are subtle differences in the results relating to the division of the default-mode network between Biswal et al. (2010) and this paper: here, the subcomponent focused on the medial prefrontal cortex (C.06) shows less activation in the posterior cingulate and parietal parts of the network than the corresponding component found by Biswal et al., while the posterior component with the main activation in the posterior cingulate cortex shows a marked coactivation in the medial prefrontal cortex, where the corresponding component found by Biswal et al. has very little coactivation. This variation in the spatial segregation of overlapping networks by spatial ICA can be attributed to methodological differences, in particular due to the fact that spatial ICA intrinsically guarantees spatial independence of components and thus enforces a more or less arbitrary delineation of borders between possibly intermingled networks. Temporal ICA might resolve this issue but, due to its computational demands and the low number of time points in most experiments, is not yet widely used in fMRI research. With current multi-band acquisition protocols and their high temporal resolution, resulting in more time points without increasing scan duration, temporal ICA becomes an increasingly viable approach (Smith et al., 2012).

Altogether, the networks identified in this study correspond well to networks already found in the literature. For example, in Damoiseaux et al. (2006), network A corresponds well to our network C.01, B to C.03, C to C.09, D to C.07, H to C.10, I to C.13, and K to C.06. Note in particular that, despite the low model order of 10, Damoiseaux et al. found a segregation of both the default-mode network and the auditory network into two subcomponents, though their split of the auditory network was different than the one identified in our study, highlighting the apparent heterogeneity in this area. As another example, Smith et al. (2009) also found components matching our components rather closely: their component 1_20_ corresponds to C.02, 2_20_ to C.01, 3_20_ to C.10, 4_20_ to C.03, 6_20_ to C.08, 7_20_ to C.13, 8_20_ to C.11, 9_20_ to C.07 and 10_20_ to C.09. Networks C.04 and C.12 have no immediate counterparts in these two studies, though component C.04 can at least to some extent be related to components in high model order studies, e.g., to component 50 in Allen et al. (2011). C.12, being among the less consistent components in our sample, might be regarded as spurious unless it can be corroborated in future studies.

On the other hand, our study did not find some components otherwise typically found in resting-state ICA studies. First, we found fewer artifactual components than most previous studies, with the lack of a WM component being the most obvious; this might be due to different preprocessing strategies. Second, we found no basal ganglia component, which has been found in many (e.g., Robinson et al., 2009; Smith et al., 2009; Biswal et al., 2010), but not all (e.g., Damoiseaux et al., 2006) resting state ICA studies. Finally, our results did not include a separate cerebellar component, and instead included some cerebellar activity into the lateralized fronto-parietal components. One reason for this might lie in the differences in field of view between studies, with different coverage of the cerebellum, but other aspects of data quality (scanner performance, noise, motion, physiological effects) also introduce variability between the data of different studies.

This between-study variability leads to one of the main limitations of the FENICA method. Since it implicitly assumes that the group components appear in every subject (the assumption lies in the fact that the best matching component of every subject is averaged for the final group component maps), the algorithm is less likely to detect components that are not present in every subject, for example a cerebellar component if the cerebellum is not wholly within the field of view of all studies included in the analysis. This is corroborated by Biswal et al. (2010) who, using a different method for the group ICA, identified a cerebellar network on data from the 1000 Functional Connectomes database. On a related note, in the short duration of a typical fMRI resting-state scan, it is possible that not all networks show a distinguishable activity pattern in all subjects to be discerned by ICA methods, which might also account for some between-subject variability in the networks identified. This fact is also a useful reminder that there are limits in the interpretability of individual ICA components (Moser and Ranjeva, 2010).

Another confounding effect could be the influence of motion on the component map estimation, as highlighted by Power et al. (2012), which could also generate some between-subject and even between-study heterogeneity if subjects of different studies differed in their head motion in the scanner. However, as Power et al. (2012) also pointed out, the motion effects they described are only of limited magnitude in adults, and a specific correction for these effects seems only necessary in studies with children or adolescents.

This work presents the largest exploratory fMRI study to date, including 1000 single subjects simultaneously, made possible due to new computational methods implemented in an R framework (Boubela et al., 2012). In Biswal et al. (2010), group ICA as the most complex of the computational tasks involved was performed separately on multiple subsets of 306 subjects due to computational limitations preventing simultaneous analysis of the whole sample. The extensive work of the R community in the handling of large datasets and parallel computing, including computation on graphics processing units (GPUs), provides the tools for analyses previously prohibitive from a computational point of view and accelerates the emergence of data driven discovery science in the field of neuroimaging. For instance, the exploratory approach used in this work allows for the unbiased drawing of an overall picture of the substantial amount of data acquired in 33 studies performed by 26 centers worldwide, while still keeping the total processing time under a week. The most important addition to the current knowledge made possible by the computational techniques employed, however, is the assessment of heterogeneity of resulting components with respect to the entire sample, both at the level of single subjects and of individual studies. As a result, an overview on networks more commonly found in individual studies as well as an assessment of divergence between the sets of networks intrinsically emerging from the data of different centers has been presented, possibly providing some guidance for the interpretation of variability in resting-state networks obtained in past and future studies. This paper shows the richness of evidence present in the 1000 Functional Connectomes dataset, but ultimately only scratches the surface of what can be examined and opens a host of new questions to be answered in future analyses.

### Conflict of interest statement

Siegfried Kasper has received grant/research support from Eli Lilly, Lundbeck, Bristol-Myers Squibb, GlaxoSmithKline, Organon, Sepracor, and Servier; has served as a consultant or on advisory boards for AstraZeneca, Bristol-Myers Squibb, GlaxoSmithKline, Eli Lilly, Lundbeck, MSD, Pfizer, Organon, Schwabe, Sepracor, Servier, Janssen, and Novartis; and has served on speakers' bureaus for AstraZeneca, BMS, Angelini, Eli Lily, Lundbeck, Schwabe, Sepracor, Servier, Pfizer, Pierre Fabre, and Janssen. All other authors declare that their research was conducted in the absence of any commercial or financial relationships that could be construed as a potential conflict of interest.
